# A Miniaturized System for Rapid, Isothermal Detection of SARS-CoV-2 in Human and Environmental Samples

**DOI:** 10.3390/biomedicines11072038

**Published:** 2023-07-20

**Authors:** Jake Staples, Athanasia-Maria Dourou, Irene Liampa, Calvin Sjaarda, Emily Moslinger, Henry Wong, Prameet M. Sheth, Stilianos Arhondakis, Ravi Prakash

**Affiliations:** 1 Department of Electronics Engineering, Carleton University, Ottawa, ON K1S 5B6, Canada; jakestaples@cmail.carleton.ca; 2 BioCoS P.C., 73100 Chania, Crete, Greece; a.dourou@biocos.gr (A.-M.D.); i.liampa@biocos.gr (I.L.); s.arhondakis@biocos.gr (S.A.); 3 Kingston Health Sciences Centre, Kingston, ON K7L 2V7, Canada; calvin.sjaarda@kingstonhsc.ca (C.S.); moslinger.emily@queensu.ca (E.M.); henry.wong@kingstonhsc.ca (H.W.); prameet.sheth@kingstonhsc.ca (P.M.S.)

**Keywords:** loop-mediated isothermal amplification, soft microfluidic chip, severe acute respiratory syndrome-coronavirus 2 (SARS-CoV-2), system on board integration

## Abstract

We report a small-footprint cost-effective isothermal rapid DNA amplification system, with integrated microfluidics for automated sample analysis and detection of SARS-CoV-2 in human and environmental samples. Our system measures low-level fluorescent signals in real-time during amplification, while maintaining the desired assay temperature on a low power, portable system footprint. A unique soft microfluidic chip design was implemented to mitigate thermocapillary effects and facilitate optical alignment for automated image capture and signal analysis. The system-on-board prototype, coupled with the LAMP primers designed by BioCoS, was sensitive enough to detect large variations in viral loads of SARS-CoV-2 corresponding to a threshold cycle range of 16 to 39. Furthermore, tested samples consisted of a broad range of viral strains and lineages identified in Canada during 2021–2022. Clinical specimens were collected and tested at the Kingston Health Science Centre using a clinically validated PCR assay, and variants were determined using whole genome sequencing.

## 1. Introduction

Loop-mediated isothermal amplification (LAMP) is a high specificity, rapid nucleic acid amplification mechanism [1]. LAMP provides faster time to results, lower complexity, and lower cost compared to Polymerase Chain Reaction (PCR) tests while providing higher accuracy than rapid antigen tests (RAT) [2,3,4,5,6]. During the present pandemic, diagnostics were shown to be crucial for an efficient response, population health surveillance, and optimization of patient management. Diagnosis was mainly based on two types of technologies: molecular and antigen. Within the former category, a variety of POC/near-POC technologies were successfully produced, accelerating the path to POC solutions. These are based on a variety of functional principles, e.g., RT-PCR, LAMP assays, streamlined nucleic acid amplification, CRISPR, etc. The most commonly used system for the detection of SARS-CoV-2 during the pandemic was RT-PCR; however, this method of detection requires significant resources and infrastructure including laboratory support and trained personnel, making it almost impossible to implement as a POC [7]. Furthermore, most commercial SARS-CoV-2 RT-PCR assays have limited data on their test performances with environmental samples.

While other nucleic acid tests like CRISPR, and nucleic acids tests like Recombinase Polymerase Amplification (RPA), have garnered attention in recent years and are suitable for portable POC integration with lateral flow assays and microfluidic devices, they have inherent challenges [8,9,10]. Established CRISPR systems are not able to quantify the amount of nucleic acid and thus can only be used as qualitative biosensors [9]. Furthermore, many CRISPR-based sensing platforms rely on isothermal amplification processes such as LAMP to increase the limit of detection, which increases their complexity [8,9]. Amplification-free CRISPR devices require further development for deployment as a POC device [11]. RPA suffers from challenges such as the tradeoff between non-specific amplification at low temperatures and complex primer and probe design, poor-user friendliness, and microfluidic channel design challenges for high viscosity reaction buffers [10,12].

The system herein presents distinct advantages compared to existing solutions: (i) reporting qualitative and quantitative results, (ii) proven better performance in environmental samples, (iii) automated signal processing, and (iv) small and cost-effective components to enhance portability and accessibility.

Accurate detection of COVID-19 remains relevant during the transition from pandemic response to control for a variety of reasons, e.g., identifying new outbreaks, re-infections, and the emergence of variants of concern (VOCs), and understanding their impact. Thus, continuous surveillance for SARS-CoV-2 not only safeguards individuals with enhanced risk of infection or the clinically vulnerable, but also ensures social safety, and enables economic recovery.

The ideal biosensor system is a fully POC device that meets the World Health Organization’s (WHO) ASSURED (affordable, sensitive, specific, user-friendly, rapid, and robust, equipment-free, and deliverable to end user) criteria [13]. A fully POC device can take a primary sample from a patient, perform nucleic acid extraction, and sample preparation by mixing the sample with the LAMP master mix, then have the system perform the nucleic acid amplification and interpret the results with high sensitivity and specificity. Many recent studies have successfully developed POC devices that rely on the naked eye to interpret qualitative assay results [14,15] or complex read-out systems to analyze the assay [16,17]. Other recent studies have used a digital camera to evaluate the result of the assay, but rely on the sample being prepared in PCR tubes prior to amplification [6,18,19], or a complex and expensive amplification process such as the use of a centrifugal device, or droplet microfluidics [20,21]. As shown by [19], artificial intelligence (AI) can be used to augment the sensitivity and specificity of LAMP assays, but it requires images to train and classify features and patterns [19]. Moving towards a POC device that uses AI to maximize the assay’s sensitivity and specificity, system alignment between the integrated camera, the laser, and the droplet becomes critical. Microfluidics can be used to maintain alignment; however, the mitigation of evaporation and thermocapillary effects becomes paramount to the design of the microfluidic channels such that the full volume of the assay has phase and spatial stability [22]. Furthermore, the variety of microfluidic techniques provides a process mechanism to integrate sample lysis and preparation, which is needed for a fully POC solution [14,16,17,20,21].

In this work, we demonstrate a miniaturized LAMP optical biosensor setup for the detection of COVID-19 gene sequences suitable for point-of-care (POC) adaptation. Two small-footprint systems were designed and tested for detecting extracted RNA of various SARS-CoV-2 strains. Moving towards a POC system, the later system required improvements in automated analysis, footprint size, and the ability to incorporate automated sample preparation. This augmented small-footprint system aligned an integrated camera and the laser with a soft microfluidic chip to achieve the desired optical alignment for automated data collection. The mitigation of evaporation and thermocapillary effects were paramount considerations in the design of the microfluidic chips such that the droplet position and size remained fixed during automated image sampling. The system used a negative template control to establish a baseline florescence threshold, and viral detection was incurred when fluorescence amplification occurred. Successful detection of clinically extracted human and environment RNA samples over a broad C_T_ range was performed.

## 2. Materials

### 2.1. Microfluidic Chip Design and Fabrication

**Figure 1:**
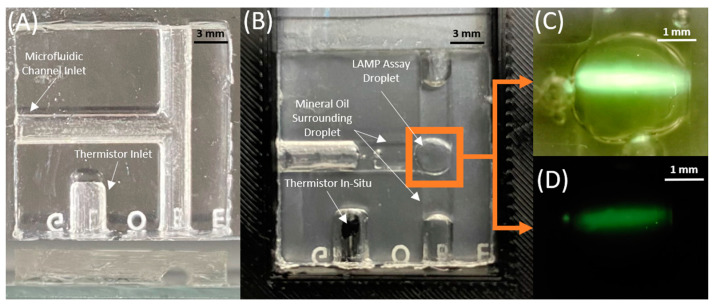
(**A**) Top and side view of the fabricated soft microfluidic chip, (**B**) soft microfluid chip loaded with sample and inserted into optically aligned biosensor with thermistor insertion, (**C**,**D**) OV5642 camera droplet capture in backlit (**C**) and low-light (**D**) conditions.

To mitigate thermocapillary effects and evaporative sample losses, a soft microfluidic device was fabricated using a Sylgard 184 silicone elastomer to suspend the LAMP assay droplet (Figure 1). Mineral oil was chosen as an immiscible carrier liquid to surround the droplet in the channel and prevent evaporation of the droplet [22]. The microfluidic channel shape was designed to minimize thermal gradients that cause inadvertent thermocapillary actuation within the microfluidic channel [22,23]. The channel ends were left open such that there is no net force on the x-y horizontal plane of the microfluidic chip caused by localized temperature gradients. Thus, the capillary effect at the channel–oil interface is relied on to prevent an outward flow of liquid. A T-shaped channel was designed to balance the thermocapillary forces at the oil–air interfaces (Figure 1A). Any existing thermal gradient was minimized by partially wetting the droplet to the surface of the capillary at the back of the T-junction [22]. The channel was designed to be small such that there is a strong capillary force between the channel and the oil, and such that the droplet is large enough in the channel to wet the back surface of the T-junction. The spherical geometry of the droplet maintains its thermal stability in the semi-cylindrical channel if such a thermal gradient were to cause thermocapillary actuation of the oil plug. The mold for the microfluidic structure was fabricated using a Prusa i3 mk3s+ 3D printer using Polyethylene terephthalate glycol (PETG) with a layer height of 0.05 mm. The structure also contains a semi-cylindrical cavity used for the insertion of a thermistor for in situ temperature measurement (Figure 1B). The microfluidic chips are prepared by mixing Sylgard 184 silicone elastomer and curing agent 1:10, then desiccated until air bubbles are completely removed. The Sylgard 184 silicone elastomer is poured inside the designed mold, then cured for 24 h. Once desiccated, it is then layered on a glass slide using a blade coater to achieve a thickness of ~100 mm. The cured microfluidic structure is inserted onto the Sylgard 184 silicone elastomer layered glass slide, then desiccated together until air bubbles are removed. The glass, the uncured Sylgard 184 silicone elastomer, and the cured microfluidic feature are then cured at 100 °C for 15 min to form the microfluidic chip.

### 2.2. Optical Assembly

**Figure 2:**
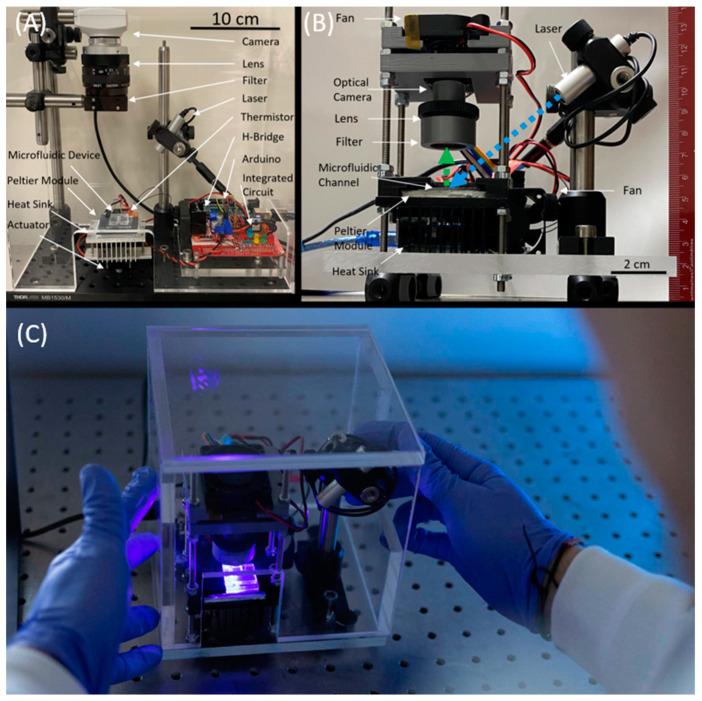
(**A**) Preliminary biosensor setup with Edmund Optics EO-0413C CMOS camera; (**B**) the proof-of-concept miniaturized biosensor with an ATmega-328p (Arduino) microcontroller-based system-on-board automated OV5642 camera, laser and temperature control; (**C**) the handheld LAMP-in-a-Box portable unit for in-field testing based on system-on-board biosensor shown in (**B**).

The thermal stability of the droplet allowed sustained alignment between the RT-LAMP droplet and the 450 nm laser (Thorlabs, Newton, MA, USA) to be captured by a CMOS camera chip (Figure 1). The soft microfluidic chip was first interfaced with an Edmund Optics EO-0413C CMOS camera, and LN298N H-Bridge regulating the Peltier Module for initial validation. For reported experimental validation, an Arducam 5 MP Plus OV5642 Mini Module SPI camera was chosen to replace the packaged, off-the-shelf CCD camera from the preliminary setup due to its small footprint, low-cost, and integrability with the ATmega-328p microcontroller, allowing us to achieve system-on-board automation of the sensor read-out (Figure 2A,B). The camera was fitted with a 1/1.8″ 4 K 12 mm low distortion M12 lens, as the 1/1.8″ (14.11 mm) optical format is large compared to the 2.74 mm sensor size of the CMOS camera, making it suitable for the low light detection of the LAMP assay droplet. The thin lens approximation of Snell’s Law was used to determine the focus plane of the image at the desired magnification. The camera could be controlled through script and user interface to capture images in both well-lit and low-light ambient conditions (Figure 1C,D). The system-on-board biosensor was packaged into the LAMP-in-a-Box setup shown in Figure 2C.

### 2.3. System-on-Board Automation for Temperature Control and Sensor Read-Out

The temperature controller was designed using an ATmega-328p (Digi-key, Thief River, MN, USA) microcontroller board which regulated a Peltier module originally via an LN298N motor driver, but was then augmented to modulation through an N-channel power MOSFET (Figure 2A,B). The temperature controller used thermistor feedback and a modified PI controller to maintain a temperature of 67 °C throughout the reaction. The system-on-board was integrated through USB with a graphics user interface (GUI), which allowed temperature setpoint control, laser control, and enabled camera capture. The camera button enabled a predefined time-lapse camera capture sequence, which while enabled, illuminated the 450 nm laser that excited a fluorescence from the droplet, which was captured and stored by the OV5642 camera as digital photographs on an SD card. The capture sequence was automatically repeated every 1 min until the end of the experiment, when the contents of the SD card were then transferred for data analysis.

## 3. Methods

### 3.1. SARS-CoV-2 Clinical Samples: PCR and Whole Genome Sequencing

Patient diagnostic samples and data were collected from the Kingston Health Sciences Center clinical laboratory. Environment samples were collected by the Coronavirus in the Urban Built Environment (CUBE) researchers in Ottawa by swabbing healthcare facilities across Ontario, Canada during 2021–2022, both before and after the emergence of the BA.2 Omicron variant. Environmental swab sample collection methods are described in [24]. The strains and viral lineages of the tested SARS-CoV-2 samples are shown in Table 1 below.

Swabs, either nasopharyngeal, nasal, and/or oral, were tested using a laboratory-developed dual-target [E-gene, 5′UTR] PCR assay that was clinically validated at KHSC [25]. Viral transport medium (VTM) from nasal samples was heat-inactivated at 65 °C for 30 min in a hot air oven. The Maxwell HT Viral TNA Kit (Promega AX2340) was used for nucleic acid extraction from 0.2 mL of VTM on the Microlab STARlet liquid handler (Hamilton Company, Reno, NV, USA). The SARs-CoV-2 diagnostic qPCR targeting the E-gene and 5′UTR was conducted on the QuantStudio 5 Real-Time PCR System (Thermofisher, Waltham, MA, USA).

Whole genome sequencing (WGS) was performed using the COVIDSeq Test library preparation kit 10 (Illumina, San Diego, CA, USA) and ARTIC V4.1 primers. Libraries were loaded at 9 pM for 2 × 150 bp sequencing on the MiSeq instrument (Illumina). Sequencing files were de-multiplexed using the native instrument software for downstream analytics. The analysis of the WGS data, including dehosting, generation of a consensus sequence, and variant calling with freebayes, were performed using a Nextflow pipeline for running the ARTIC field bioinformatics tools [26] (https://github.com/jts/ncov2019-artic-nf). Viral lineages were assigned using pangolin version 4.06, Scorpio version 0.3.17, and constellations version v0.1.9. Data was analyzed using PRISM 9.0 and R Studio.

### 3.2. LAMP Primer Design

The novel SARS-CoV-2 LAMP primers were designed by BioCoS P.C. using its innovative bioinformatics tool; the Biomarkers Computational System [27]. This proprietary tool facilitates the rapid and accurate identification of novel biomarkers (genomic loci) with high species-specificity, while it reduces the need for de novo sequencing and bypasses whole genome alignments. In this work, the tool’s capacity has been expanded from past applications in the food sector to analyzing genomic data for a variety of viral pathogenic strains. The unique LAMP primers, reported in Table 2, were designed upon a novel biomarker with 245 bases in length at the ORF1ab gene; this was identified in late 2020 on the SARS-CoV-2 isolate Wuhan-Hu-1 (NC_045512.2). This discovery occurred before the emergence of new strains, and it included the processing of a dataset with 10,011 non-SARS-CoV-2 viral genomes. Furthermore, this novel biomarker selectively detected SARS-CoV-2 RNA regardless of lineage specificity, and was in silico validated using BLAST (blastn) queries that compared the sequence with viral, bacterial, and human genomes, as well as the extensive and updated betacorona virus database. The results verified the biomarker’s high specificity, and the sequence was subsequently input to the PrimerExplorer V5 online tool for LAMP primers design [28]. The selection of the best candidate LAMP pair was based on the process described by Notomi et al. 2015. The selection algorithm includes rules that ensure stability at the end of primers, attaining a GC nucleotide content between 50% and 60%, and limiting complementarity at the 3′ end [29]. The selected primer sequences were synthesized by Integrated DNA Technologies (IDT, San Diego, CA, USA).

### 3.3. Experimental Procedure

LAMP assay solutions were prepared by aliquoting 12.5 µL of WarmStart LAMP Master Mix (New England Biolabs, Whitby, ON, Canada), 0.5 µL of fluorescent dye (New England Biolabs, Whitby, ON, Canada), 2.5 µL of novel LAMP primers and probe mixture (100 nL FIP, 100 nL BIP, 12.5 nL F3, and 12.5 nL B3 in 2.275 µL nuclease-free water), and 7 µL of nuclease free water. 2.5 µL of SARS-CoV-2 RNA from extracted clinical and environmental samples was added to the mix, 12.5 µL of the 25 µL mix was aliquoted into the oil pre-filled microfluidic chip, and the assay droplet was positioned at the T-junction of the microfluidic chip.

### 3.4. Real-Time Detection and Image Analysis

**Figure 3:**
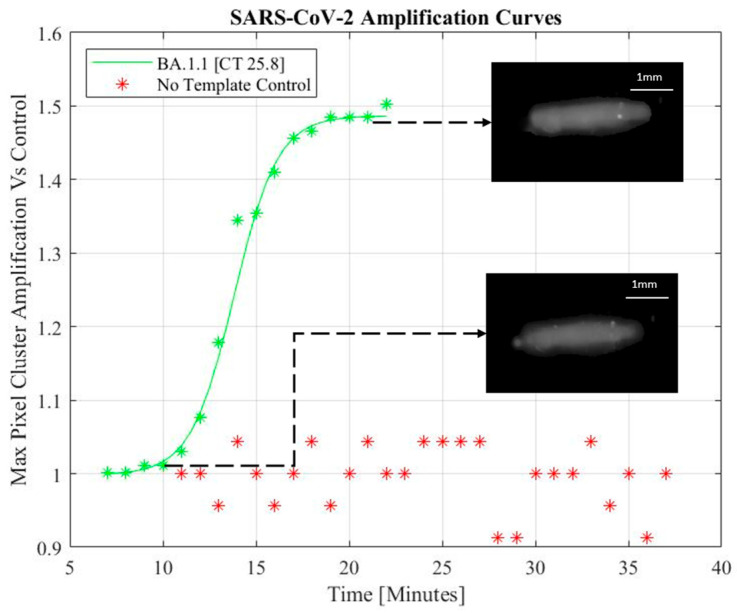
SARS-CoV-2 fitted amplification curve (solid) and experimental data (green Asterisk) with NTC data processed from low-cost setup.

In the preliminary experiments, images were captured using a packaged CCD camera (see Figure 2A), with commercial software able to monitor real-time maximum pixel intensities to be recorded and plotted as an exponential increase in detected photocurrent. In the miniaturized automated setup, an automated sequence of images is taken using the Arducam 5MP Plus OV5642 Mini Module SPI Camera at an interval following the initial amplification period (Figure 3). Once stored, these images were imported to MATLAB for automated analysis and curve generation of the maximum pixel cluster. The MATLAB algorithm selected only the green components of the RGB image, then used a morphological grayscale opening with a circular structuring element of a radius of 4 pixels. A grayscale opening performs an erosion, then dilation, of the image. The erosion takes each pixel in the image and represents the pixel by the minimum pixel value in the neighborhood contained by the structuring element. The dilation then follows, and each pixel in the image is represented by the new maximum value present in the neighborhood of the structural element. Overall, the opening represents finding the maximum of the minimum value within the neighborhood defined by the structuring element, effectively smoothening the pixel intensities at the boundary of the fluorescing droplet. This eliminated noisy pixels from each photo and provided a distinct boundary between the foreground and the background of the droplet. The Otsu thresholding technique is then used to identify the florescent droplet against the background. A grayscale opening was again used with a circular structuring element of a radius of 4 pixels to smoothen the pixels of the binarized droplet. The maximum pixel intensity of the droplet representing the brightest pixel cluster, and the total fluorescence emitted by the droplet, is calculated, and recorded for each captured image. Data was recorded in an array corresponding to the time-lapse sequence, and a plot of these two values was generated as the images were processed. The arrayed data was fit to a sigmoidal curve by customizing MATLAB’s fittype function to the form of a sigmoid and using the least absolute residual robust (LAR) method built into MATLAB’s fit function.

## 4. Results and Discussion

### 4.1. Real-Time Detection of Clinical and Environmental Samples

A miniaturized microfluidic biosensor using LAMP is demonstrated as a small-footprint rapid molecular test system. An exponential increase in the photocurrent indicated detection of SARS-CoV-2. The addition of image processing to the automated setup allowed for the mitigation of noisy pixels. Sigmoidal functions were used to model the maximum pixel intensity amplification of the processed images captured in one-minute intervals following an initial annealing period. The pixel intensities were normalized to the fluorescence baseline established by the first image captured. Despite the low-cost camera, a 4X smaller footprint than the previous design, and a lowering of the droplet’s fluorescence Intensities due to the grayscale opening operation, the augmented system’s microfluidic droplet alignment and image analysis was able to generate qualitative amplification curves indicative of positive SARS-CoV-2 assays. Figure 3 shows the sigmoidal model through the data of a positive LAMP sample, while a negative template control sample (NTC) experiences no detectable signal amplification.

### 4.2. Overall Performance of LAMP Based Miniaturized Platform

**Figure 4:**
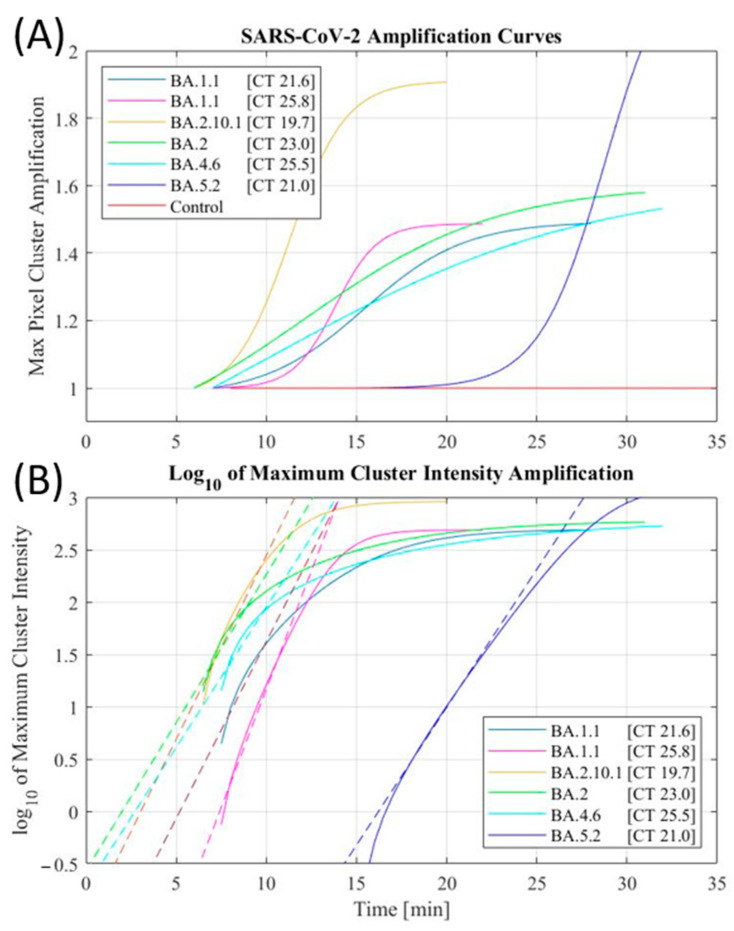
Fitted amplification curves tracking maximum pixel cluster (**A**), and logarithmic fitted amplification curves (solid) with regressions in the linear regions (dashed) of the logarithmic curves (**B**).

Our novel LAMP primers successfully detected several emerging SARS-CoV-2 strains in both clinical and environmental extracted RNA samples. Both the preliminary and augmented automated setups were effective in the detection of SARS-CoV-2 extracted RNA. These curves were extracted from the data of many samples (Figure 4A). Furthermore, to determine the release of fluorescent dye by a positive reaction, the summation of foreground pixel intensities was also performed to evaluate the increase in fluorescence throughout the droplet. The summation of fluorescence of the droplet followed similar trends to the maximum intensity tracking, without the limitations set by the grayscale opening operation. Sigmoidal curves were also able to model the summed fluorescence reliably, while the no-template control sample showed a negligible increase in total fluorescence from its initial annealing through the entire amplification period.

**Figure 5:**
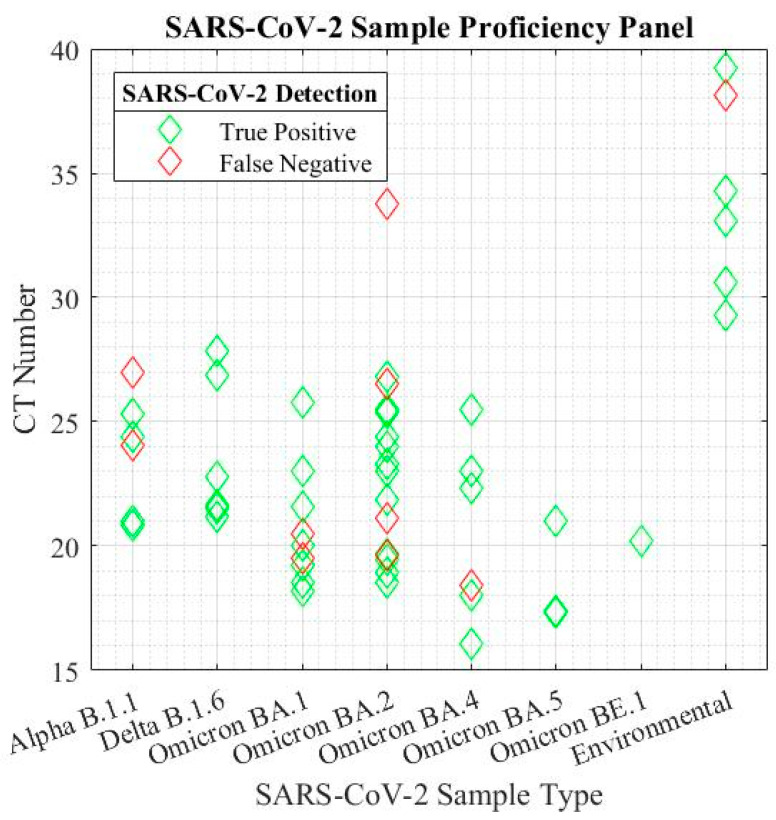
Proficiency panel of biosensor sensitivity across SARS-Cov-2 strains in human and environmental samples as reported in Table 1.

The sensitivity of the system was verified by strain and viral load. The sensor produced an overall sensitivity of 81.48% across the 54 tested samples, and the sensitivity of the biosensor was not affected by new strains of SARS-CoV-2. Cyclic threshold (CT) values were verified using a benchtop RT-PCR system, and the sensitivity was determined in a proficiency panel across all strains of SARS-CoV-2 (Figure 5). The shorthand notation for human-collected viral lineage samples represents a multitude of SARS-CoV-2 strains in both human and environmental samples. The full viral lineages are detailed in Table 1 of the Materials section. The environmental data represents a combination of lineages collected in healthcare facilities across Ontario, Canada before and after the emergence of the BA.2 omicron variant.

Reliable detection was established using both the maximum pixel cluster intensity amplification and the summation amplification of the total fluorescence. The sigmoidal fits of these amplification curves were also considered when determining the outcomes of the reactions. The logarithmic plot of the maximum pixel cluster (Figure 4B) displays a linear fit to the positive exponential portion of the sigmoidal, which appears as a linear slope in the logarithmic curve. Extraction of the x-intercept of the logarithmically transformed plots corresponds to the time to amplification, like the cycle number in an RT-PCR system. The anticipated time of amplification was expected to mirror the CT values of the samples; however, due to the low-cost nature of the setup, perfect RT-PCR curves were not achieved in all circumstances. Thus, the extracted shape of the curve is used as supporting evidence, along with maximum pixel amplification and summed fluorescence amplification, to evaluate the detection of SARS-CoV-2 in the performed assays.

### 4.3. Current Capabilities and Limitations

The low-cost system-on-board integrated portable LAMP biosensor offers many benefits towards POC diagnostics, pandemic surveillance, and accessibility in remote, resource-limited test settings. However, the low-cost components in the miniaturized setup place limitations on captured signal quality and signal-to-background ratio. The low-cost camera assigns a broad spectrum of wavelengths contributing to red, green, and blue signal components despite physical filtration with an optical bandpass filter. Further limitations included the automated setup, which sampled the droplet by only illuminating a portion of the droplet with the laser upon image capture by the camera. The image capture and analysis therefore rely on the LAMP assay droplet fluorescing homogenously. These qualities are limitations towards achieving a quantitative LAMP bioassay; however, in the qualitative system demonstrated, these limitations can be mitigated, and a high sensitivity of detection can be maintained. The shelf life for the open channel Sylgard 184 silicone elastomer microfluidic device is greater than six months, and the dominant failure mode is delamination of the silicone 3D structure from the silicone coated glass surface due to de-gassing and ingress over a long period of time. The bioinformatics approach used to develop novel LAMP primers has been able to detect several SARS-CoV-2 viral lineages between 2020–2022; however, despite the wide spectrum of variants detected, novel primers design is a continuous process in order to incorporate further viral mutations and resultant challenges to the LAMP assay [30].

## 5. Conclusions

We have demonstrated a portable LAMP biosensor system which incorporates unique LAMP primers designed in-house, suitable to be developed into a POC rapid molecular assay platform. The system was validated using more than 50 clinical and environmental RNA extract samples consisting of several SARS-CoV-2 viral strains detected from 2021–2022. The novel LAMP primers, which were designed to detect a broad range of SARS-CoV-2 viral strains and lineages, were highly effective in detecting samples containing these strains. The system-on-board integrated imaging system proved capable of analyzing low-level fluorescence signal in samples with CT values from 16–39 using a low-cost board integrable camera, and impressively detected high CT environmental swab samples. The system captured time-lapsed images of the thermally stable assay droplet and processed the images upon completion of the amplification for the end point detection of the virus. Further augmentations to the system remain to be carried out for improved sensitivity and POC applicability. These augmentations include the addition of on-board computational power to allow for real-time image processing by the LAMP biosensor, coupled with AI-assisted detection to improve sensitivity and reduce assay time. In addition, coupling of additional laser targets will yield a quantitative, multiplexed LAMP biosensor. which would be more suitable for clinical studies. The authors are investigating a passive microfluidics-based electromagnetic nucleic acid extraction and purification system that can be modularly added to the current microfluidic chip for POC readiness in future applications. This would provide the next step towards a portable, user-friendly, POC-ready LAMP biosensor, addressing the need for the cost-effective, rapid, highly specific, and sensitive detection of pathogens.

## Figures and Tables

**Table 1:** List of viral strains and lineages of SARS-CoV-2 tested in human and environmental samples.

Strain	Lineage	CT Value	Result
Alpha B.1.1	B.1.1.7	20.85, 20.98, 24.04, 24.37, 25.3, 27.0	4/6
Delta B.1.6	B.1.617.2	21.19, 21.49, 21.65, 22.81, 26.89, 27.87	6/6
Omicron BA.1	BA.1.1	21.60, 23.00, 25.82	3/3
Omicron BA.1	BA.1	Seven samples with CT in 18–21	5/7
Omicron BA.2	BA.2	18.54, 18.94, 19.45, 19.6, 19.67, 21.84, 22.98, 23.31, 23.97, 24.4, 25.37, 25.51, 26.54, 26.82, 33.77	12/15
Omicron BA.2	BA.2.10.1	19.71	1/1
Omicron BA.4	BA.4	22.26	1/1
Omicron BA.4	BA.4.1	16.04, 17.99, 18.39, 23.00	3/4
Omicron BA.4	BA.4.6	25.49	1/1
Omicron BA.5	BA.5.1.1	17.31	1/1
Omicron BA.5	BA.5.2	17.40	1/1
Omicron BA.5	BA.5.2	21.01	1/1
Omicron BE.1	BE.1.1	20.22	1/1
Environmental Swab Samples	Mixed Samples, lineage unknown. (Swabbed between Feb. 2021–Feb. 2022)	29.26, 30.6, 33.06, 34.29, 38.13, 39.2	5/6

**Table 2:** LAMP Primers designed using the BioCoS tool and PrimerExplorer software.

LAMP	Primer Sequences
BIP	ACT TTC TGT TTT GCT TTC CAT GCA GTT GTA AGG TTG CCC TGT T
FIP	CCA TTT TTT CAA AGG CTT CAG TAG TTC ATC TAA ATT GTG GGC TCA
B3	ATG GAA GGG AAC TAA ACT CT
F3	GTT TTG CAA CAA CTC AGA CT

## Data Availability

The data presented in this study are available on request from the corresponding author.
